# Dual threshold input receiver FPGA-only signal digitization method for time-of-flight positron emission tomography

**DOI:** 10.1007/s13534-024-00380-5

**Published:** 2024-05-04

**Authors:** Guen Bae Ko, Jae Sung Lee

**Affiliations:** 1 Brightonix Imaging Inc., Seoul, 04782 South Korea; 2 Institute of Radiation Medicine, Medical Research Center, Seoul National University, Seoul, 03080 South Korea; 3 Department of Nuclear Medicine, College of Medicine, Seoul National University, Seoul, 03080 South Korea; 4 Interdisciplinary Program in Bioengineering, College of Engineering, Seoul National University, Seoul, 03080 South Korea

**Keywords:** Positron emission tomography, Field-programmable gate array, Silicon photomultiplier, Time-to-digital converter, Time-over-threshold

## Abstract

As silicon photomultiplier (SiPM)-based time-of-flight (TOF) positron emission tomography (PET) becomes popular, the need for sophisticated PET data acquisition (DAQ) systems is increasing. One promising solution to this challenge is the adoption of a field-programmable gate array (FPGA)-only signal digitization method. In this paper, we propose a new approach to efficiently implement an FPGA-only digitizer. We configured the input/output (IO) port of the FPGA to function as a dual-threshold voltage comparator through the use of simple passive circuitry and heterogeneous IO standards. This configuration overcomes the limitations of existing methods by allowing different threshold voltages for adjacent IO pins, effectively reducing routing complexity and lowering manufacturing costs. An FPGA-only digitizer was implemented by integrating the dual-threshold voltage comparator and FPGA-based time-to-digital converter. By combining the dual-threshold time-over-threshold (TOT) method and curve fitting, precise energy information could be obtained. The performance of the FPGA-only digitizer was assessed using a detector setup comprising a 3 × 3 × 20 mm^3^ LYSO scintillation crystal and a single pixel SiPM. Using the configured evaluation setup, an energy resolution of 12.5% and a time resolution of 146 ± 9 ps were achieved for a 20 mm scintillation crystal. The dual-threshold TOT implemented using the proposed method showed consistent linearity across an energy range of 100 keV to 600 keV. The proposed method is well-suited for the development of cost-effective DAQ systems in highly integrated TOF PET systems.

## Introduction

The introduction of silicon photomultiplier (SiPM) has marked a significant advancement in positron emission tomography (PET) technology [1–5]. Specifically, its fast photon response time and compact pixel size are the key attributes for the development of high-resolution time-of-flight (TOF) PET [6, 7]. However, the increased number of signal channels due to the small pixel size of SiPM has amplified the complexity of the data processing circuitry. Therefore, the need for high-performance multi-channel data acquisition (DAQ) systems for PET data processing has recently increased. One of the important performance specifications required for such a DAQ system is its ability to measure time in the range of tens of picoseconds.

To meet these technological demands, various application-specific integrated circuits (ASICs) have been developed [8–11]. These ASICs are significantly advanced over traditional bulk electronics, offering reduced power consumption per channel and enhanced space efficiency. One of their key benefits is the facilitation of individual SiPM channel signal acquisition without signal multiplexing, thereby maximizing detector performance. However, the relatively long dead-time per channel (~ several microseconds) inherent in most of ASICs can potentially degrade counting rate performance in multiplexed detectors. Additionally, the inherent limitations of ASICs, particularly their lack of post-programming capability, result in diminished flexibility. This constraint limits their suitability for specialized detector concepts, such as those requiring the analysis of scintillation signal shape.

Despite the advantageous characteristics of ASICs, field-programmable gate array (FPGA) emerges as viable alternatives due to their development flexibility and excellent performance facilitated by the latest semiconductor production process. Specifically, by implementing a time-to-digital converter (TDC) using the digital elements of an FPGA [12–17], leveraging its input/output (IO) interface for high-speed digital communication as a comparator, and applying time-based energy acquisition methods such as time-over-threshold (TOT), it becomes feasible to construct a DAQ system solely with the FPGA. This approach eliminates the necessity for additional analog-to-digital conversion circuitry such as analog-to-digital converter (ADC) and comparator. The latest FPGA-based TDCs have achieved an accuracy of less than 10 ps [18, 19]. For energy linearity, a drawback of the time-based energy acquisition method, various ideas such as multi-voltage-threshold (MVT) [20, 21] or sawtooth threshold sampling (STS) [22] have been proposed, allowing performance close to that of ADCs.

There are two approaches to substitute a voltage comparator with the FPGA's input receiver. The first method involves configuring it with a digital differential input receiver, such as low-voltage differential signaling (LVDS) [20, 21, 23, 24]. However, this technique requires two IO pins for configuring a single comparator. The alternative method employs a single-ended memory interface (SeMI), specifically designed for high-speed memory communication in the FPGA [25–27]. In this method, only one IO pin is required to implement one comparator, but it comes with a limitation that a single threshold can only be applied to all IO pins within the same IO bank (IOB).

In this paper, we introduce a novel approach that enables the application of multiple thresholds to IO pins within a single IOB while configuring single-ended input receiver. This method effectively conserves the number of IO pins required on the FPGA and aids in lowering printed circuit board (PCB) manufacturing costs by reducing the routing complexity in the PCB layout. Employing this technique, we demonstrate the concept of an FPGA-only digitizer and have evaluated its performance using a one-to-one coupled TOF detector. This approach will be highly beneficial in the development of cost-effective DAQ systems for PET and radiation detection applications.

## Methods

### SeMI with adjustable input offset voltage

**Fig. 1:**
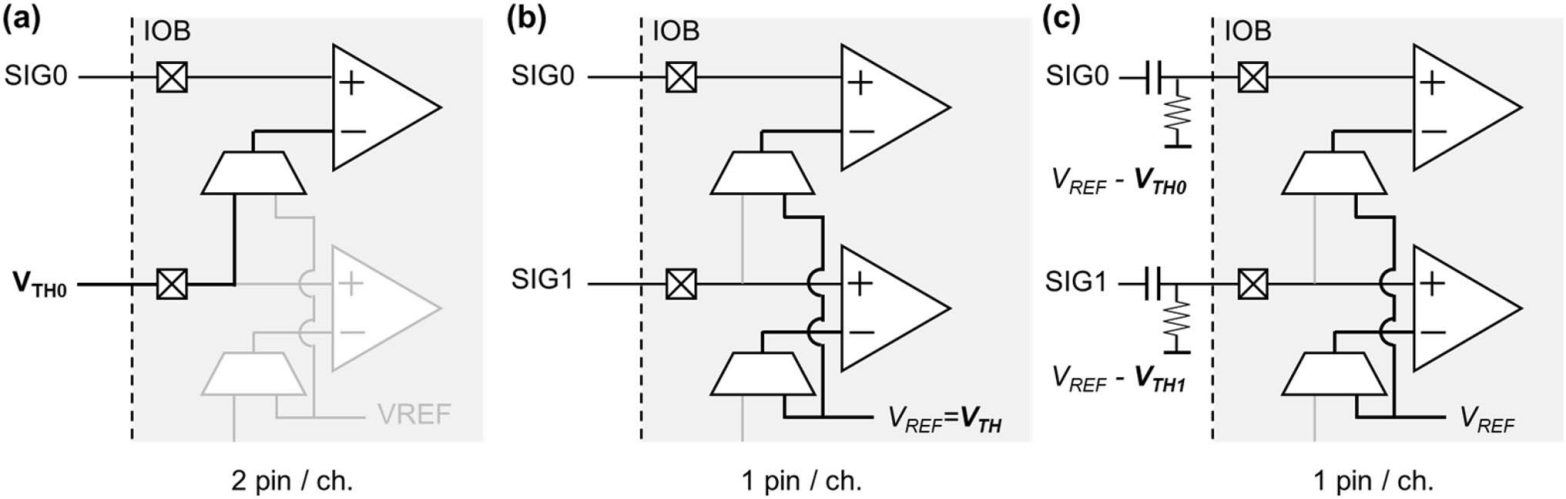
Illustration of the utilization of FPGA input receiver as comparators. **a** Differential input receivers such as LVDS, **b** single-ended input receiver such as SSTL/HSTL, and **c** proposed method

Figure 1 illustrates a previously proposed method utilizing a differential input receiver (Fig. 1a) and the SeMI technique, which employs stub serial termination logic (SSTL) and high-speed transceiver logic (HSTL) interfaces (Fig. 1b). SSTL and HSTL are the single-ended input interface designed for communication with high-speed memory systems, such as double data rate synchronous dynamic random-access memory and are available in available in modern FPGAs. In XILINX's FPGA, the reference voltage (*V*_*REF*_, threshold voltage of input receiver) used for SSTL and HSTL can be supplied externally via VREF pin specifically assigned to IOB (EXTERNAL_VREF) or internally generated in the FPGA fabric (INTERNAL_VREF), which include options like 0.6, 0.675, 0.75, 0.9 V for Xilinx 7 series FPGA. Additionally, there is a limitation that only one *V*_*REF*_ value can be used for all input receivers located in the same IOB.

Due to these implementation constraints associated with SSTL/HSTL, Won and Lee's original method presents several practical limitations. Firstly, the FPGA's INTERNAL_VREF typically exceeds 0.6 V, which is too high to effectively trigger SiPMs in TOF PET applications. As a result, the use of EXTERNAL_VREF becomes necessary, requiring the allocation of two additional IO pins per IOB for supplying external reference voltage. Secondly, the inability to apply different threshold voltages to individual pins within the same IOB restricts the optimization of threshold voltages for each detector in multi-channel implementations. Lastly, the impracticality of applying a threshold when the input signal's voltage falls below 0 mV (indicating negative polarity) confines this approach to signals with positive polarity.

To overcome these shortcomings, we applied an input offset voltage (*V*_*IOFFSET*_) using a simple resistor and capacitor at the input receiver (Fig. 1c). This technique offers the advantage of allowing for disparate offsets for each sensor or amplifier, facilitating the identification of the optimal threshold voltage. By setting an appropriate *V*_*IOFFSET*_, the INTERNAL_VREF can be used in the proposed method. Moreover, when the *V*_*IOFFSET*_ is sufficiently high, it becomes applicable to waveforms exhibiting both positive and negative polarity.

In the original SeMI approach, the external reference voltage serves as the threshold voltage (*V*_*TH*_ = *V*_*REF*_). However, in the proposed method, the threshold voltage is determined by the difference between the reference voltage and input offset voltage (*V*_*TH*_ = *V*_*REF*_*—V*_*IOFFSET*_).

### Dual threshold implementation

**Fig. 2:**
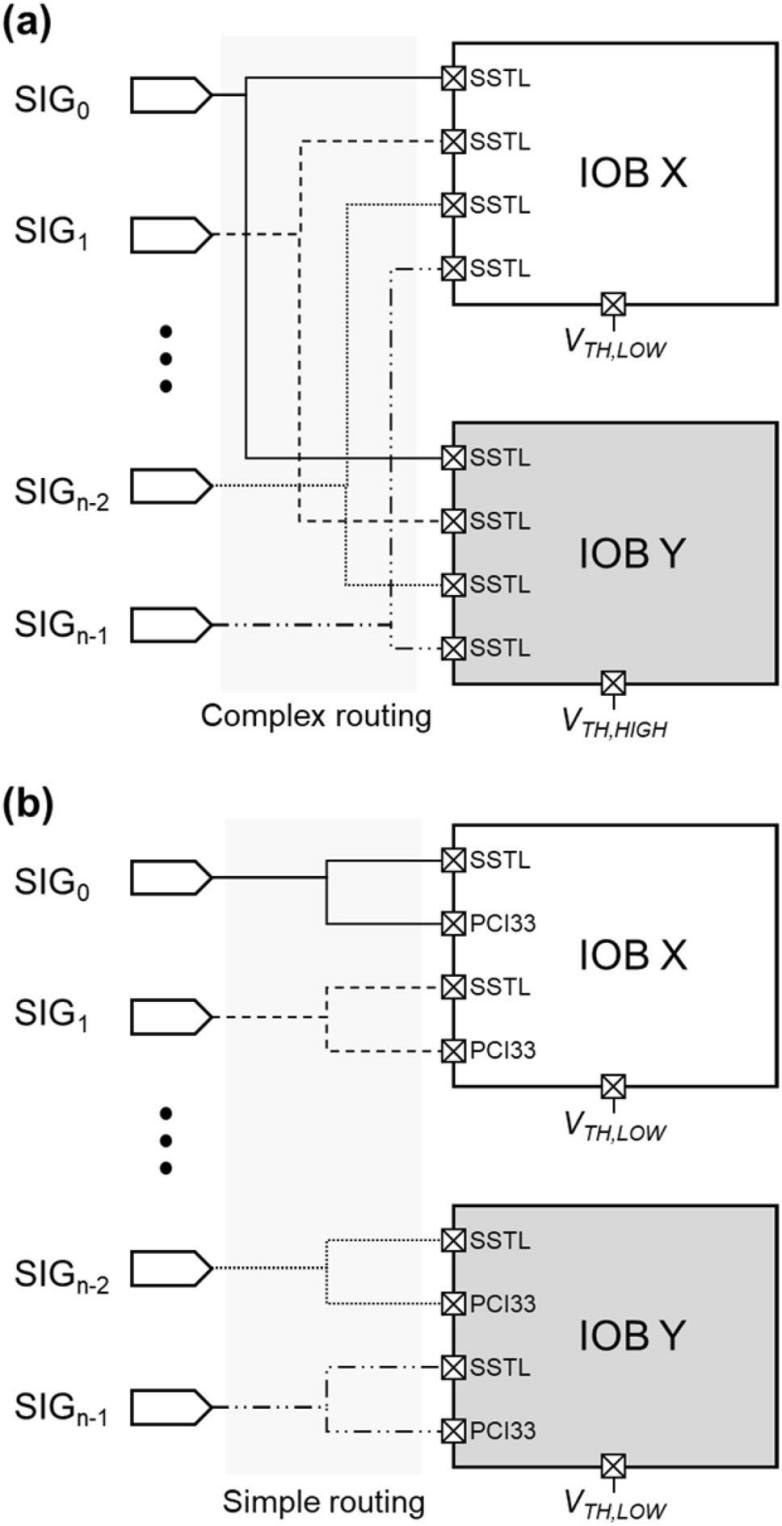
Illustration of routing complexity for **a** existing SeMI method, and **b** proposed method

In addition to apply the offset voltage at the input terminal, we propose a method to apply different *V*_*REF*_ in a same IOB. In the SeMI method, employing two distinct threshold voltages necessitates the use of two separate IOBs with different reference voltages. This setup complicates the PCB design because the same signals must be branched to different IOBs at relatively distant locations (Fig. 2a).

The proposed approach, called single-ended heterogeneous interface (SeHI), combines different types of single-ended IO standards to set different threshold voltages on the IO pins in the same IOB. Specifically, we applied SSTL/HSTL to one IO pin, while adopting other defined IO standards such LVCMOS or PCI33 for another IO pin. When utilizing digital input receivers such as LVCMOS or PCI33, a fixed reference voltage is applied internally within the FPGA, which is different from the reference voltage used by SSTL/HSTL. As a result, this configuration allows two adjacent IO pins in the same bank to have different threshold voltages (Fig. 2b, for example, SSTL/HSTL on one and PCI33 on the other). This method effectively reduces routing complexity by enabling the connection of the same signal lines to adjacent IO pins. Consequently, it lowers the number of layers needed for PCB construction, leading to reduced manufacturing costs. This approach is especially advantageous for mitigating crosstalk and minimizing induced electrical noise among signal paths.

We verified the feasibility of the proposed method using XILINX's Artix-7 FPGA (XC7A75T-1CSG324C). The supply voltage (VCCO) of the IOB used was set to 2.5 V. Unlike SSTL/HSTL IO standards where the reference voltage is set to an exact internal or external voltage, the reference voltage in LVCMOS or PCI33 is VCCO dependent and is not explicitly defined by the manufacturer. Therefore, we determined the reference voltage of LVCMOS and PCI33 under the set conditions (VCCO = 2.5 V) through the experiments.

The reference voltage serves as the threshold that digitally distinguishes between logic high (1) and logic low (0) signals. By supplying the same voltage as the reference voltage to the IO pin, we ensure that the FPGA recognizes both states, 1 and 0, equally. Leveraging this principle, we conducted an experiment where we varied the voltage input to the IO pin from 1 to 1.3 V in increments of 0.01 V. At each voltage level, we measured the ratio at which the FPGA recognized the input of the IO pin as logic high. We also conducted a reverse process, adjusting the voltage from 1.3 V back to 1 V, to observe any hysteresis effects. The reference voltage was identified at the point where ratio recognized as logic high reached 0.5.

### DAQ implementation

**Fig. 3:**
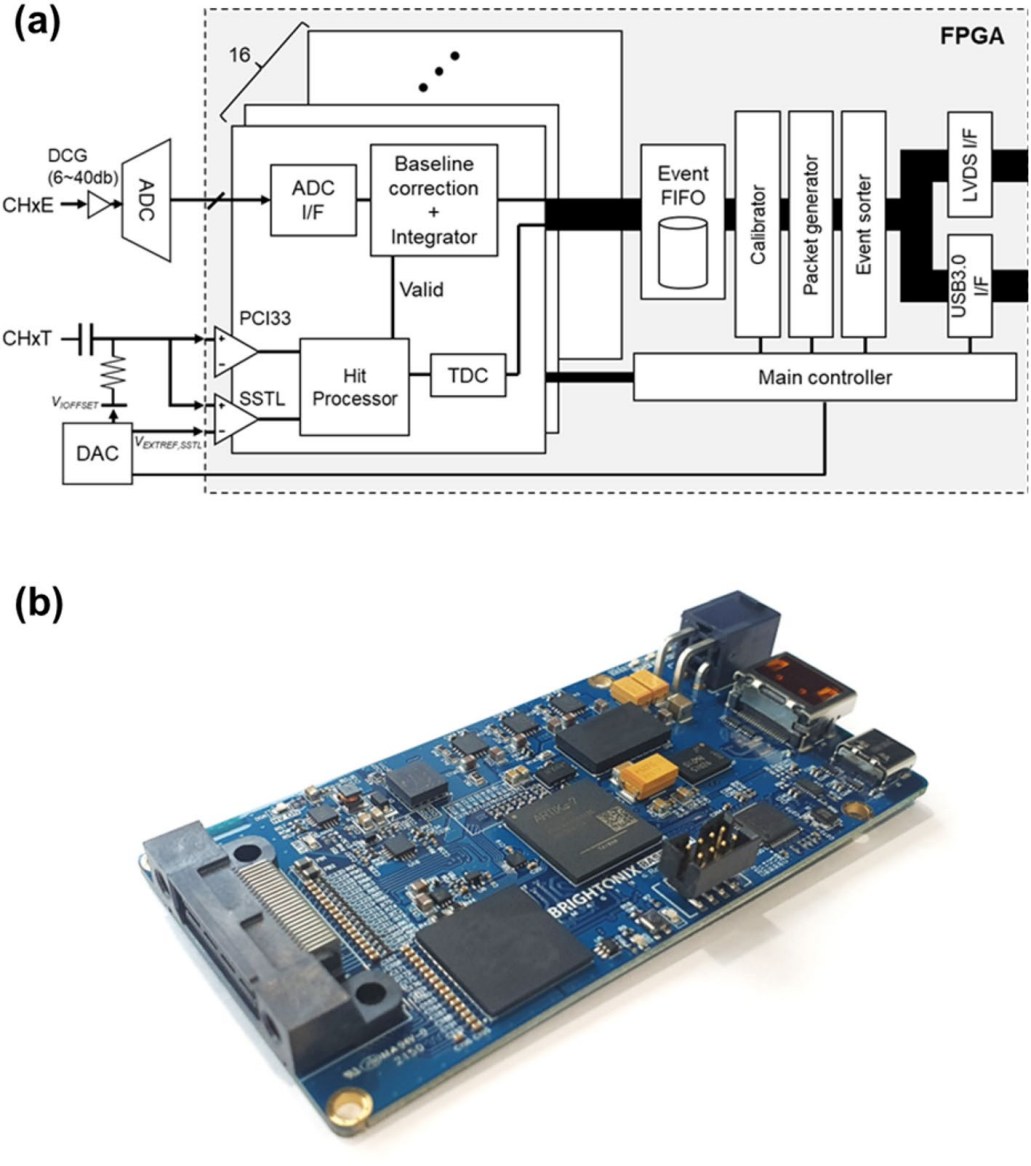
FPGA-based digitizer implemented for proof of concept. **a** Schematic and **b** photograph

We developed a cost-effective multi-channel DAQ board (BASP-10011; Brightonix Imaging Inc., South Korea) by applying proposed technology to demonstrate the concept of an FPGA-only digitizer in PET application (Fig. 3). This board is designed to process 16 energy channels (Ch0E—Ch15E) and 16 timing channels (Ch0T—Ch15T). The signal fed into the energy channel undergoes sampling by an 80 MHz free-running ADC, which is subsequently subjected to baseline correction and integration processes to yield precise energy information.

For timing channels, an RC circuit was used to set the input offset voltage (*V*_*IOFFSET*_). Each timing channel input is connected to a pair of adjacent IO pins within the same IOB. These IO pins are configured to comply with SSTL and PCI33 IO standards, respectively. The low and high thresholds are adjustable based on the external reference voltage for SSTL (*V*_*REF,SSTL*_, supplied by the VREF pin), reference voltage applied to the PCI33 IO standard (*V*_*REF,PCI33*_, determined experimentally via logic high ratio test) and *V*_*IOFFSET*_, setting the threshold according to the following equation.

1 $${V}_{TH,LOW}={V}_{REF,SSTL}-{V}_{IOFFSET}$$

2 $${V}_{TH,HIGH}={V}_{REF,PCI33}-{V}_{IOFFSET}$$

The *V*_*IOFFSET*_ and *V*_*REF,SSTL*_ were precisely controlled by the 16-bit digital-to-analog converter.

**Fig. 4:**
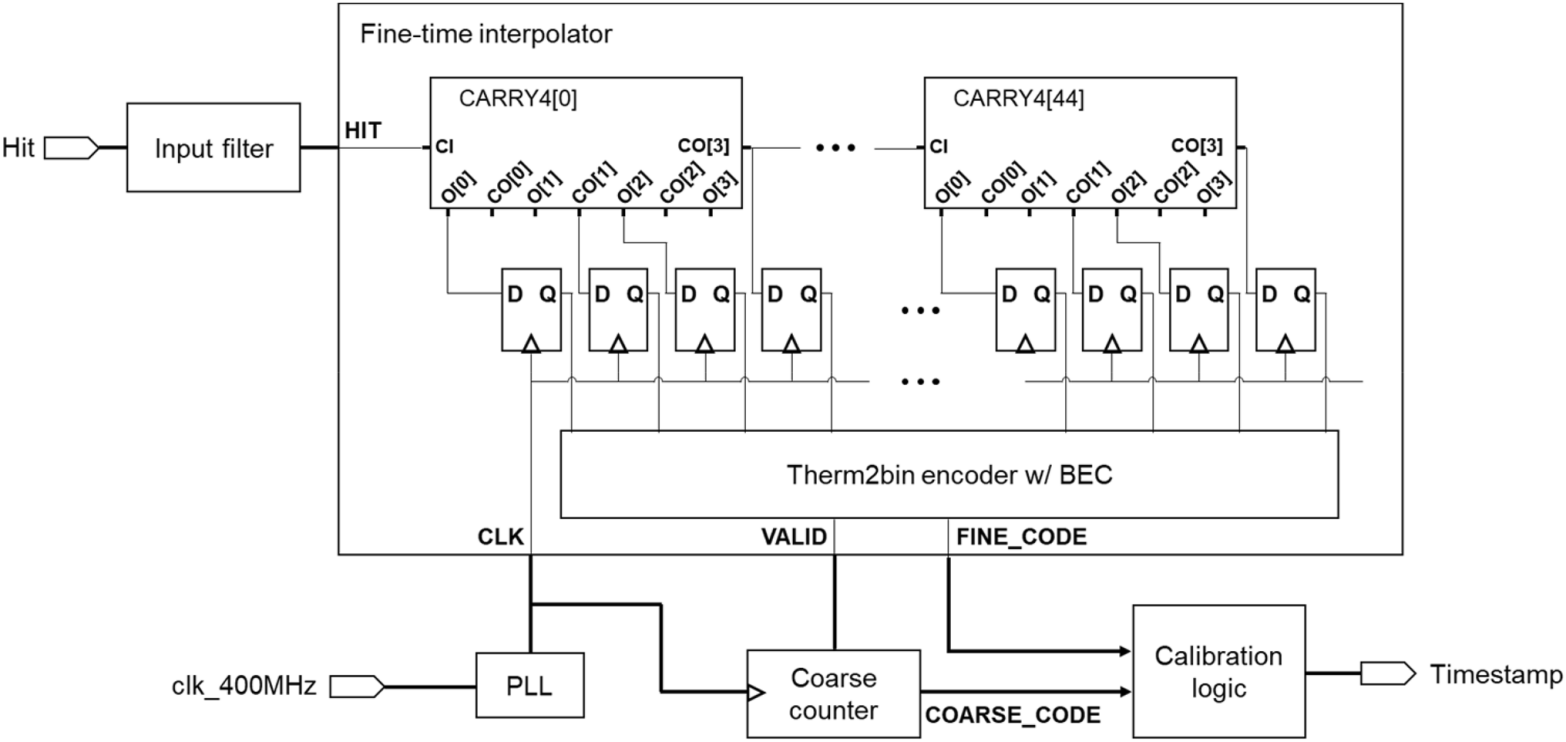
Schematic drawing of TDL-based TDC

The incoming signal to the timing channel is converted into a digital signal by the input receivers with different threshold voltage, then transferred to the time-to-digital converter (TDC). To achieve accurate time information, the FPGA-based TDC was developed using a tapped delay line (TDL). The TDL was implemented using CARRY4 logic, resulting in a total of 180 taps formed by 45 CARRY4 logic elements (Fig. 4). Both sum (O) and carry-out (CO) output of the CARRY4 is captured through a flip-flop to improve differential nonlinearity (DNL) and integral nonlinearity (INL) performance [16]. Subsequently, the output thermometer code from flip-flop is transformed into binary code by a thermometer-to-binary converter with an integrated bit error correction function, generating a fine code value. The fine code and coarse count, produced by a counter operating at 400 MHz, are converted to timestamp, with a least significant bit (LSB) of 16 ps, through calibration logic.

The collected time and energy information is organized into packets using a first-in, first-out (FIFO) buffer. Following data processing, these packets can then be transmitted to a workstation using the USB 3.0 protocol or to a signal multiplexing board for channel expansion through a customized LVDS protocol.

The logic design, implemented on the XC7A75T-1CSG324C FPGA, utilized the following resources: 40,754 Flip-Flops (FFs), or 43.17% of the total FFs available; 31,435 Look-Up Tables (LUTs), representing 66.6% of the FPGA's LUT capacity; and 135 pins, accounting for 64.29% of the total pins.

### TDC performance evaluation

To evaluate the intrinsic performance of the implemented FPGA based TDC, identical temporally random digital pulses with rise time 1 ns and pulse height 0.5 V were applied to all 16 timing channels. The intrinsic timing uncertainty of the TDC was measured by obtaining the time resolution for different TDC pairs.

### Detector performance evaluation

**Fig. 5:**
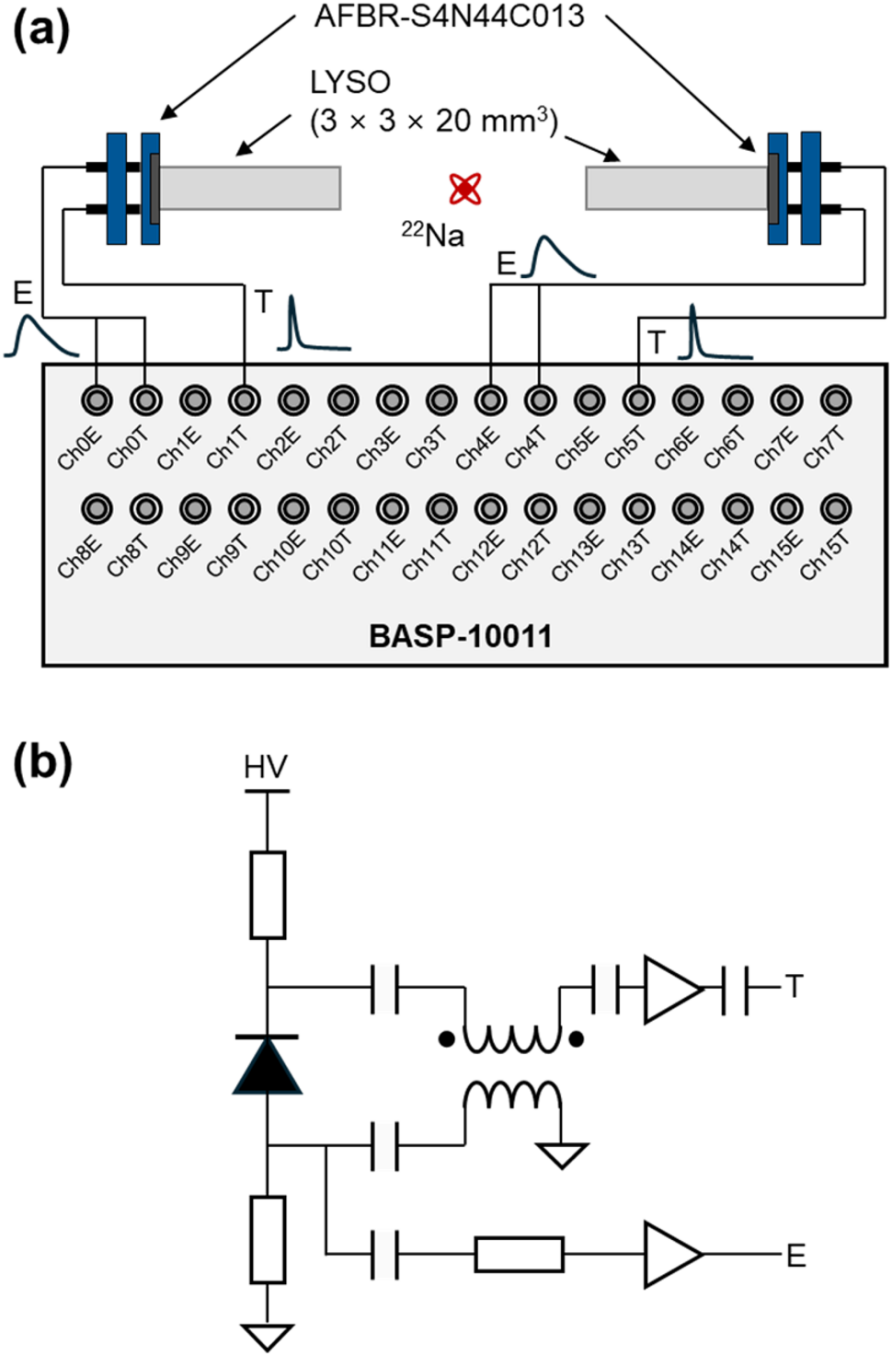
**a** Experimental setup for PET detector performance evaluation. **b** Configuration of amplifier circuit to optimize detector performance

Figure 5a shows an experimental setup to assess the feasibility of a cost-effective FPGA-only digitizer for TOF PET applications using proposed method. Two identical one-to-one coupled detectors were assembled for coincidence data acquisition. These detectors featured polished LYSO (Lu_1.9_Y_0.1_SiO_4_:Ce; EPIC crystal, China) crystals with 3 × 3 × 20 mm^3^ dimension. The crystals were enveloped enhanced specular reflectors (ESR; 3 M, USA) on five sides, leaving one surface as the light exit surface. The crystals were optically coupled to single-channel SiPMs with an active area of 3.72 × 3.72 mm^2^ and 30 × 30 μm^2^ microcells (AFBR-S4N44C013; Broadcom, USA), featuring a total of 15,060 microcells and a fill factor of 76%. The bias voltage for the SiPMs was set at 36 V.

To optimize timing performance, high-frequency timing signals were generated using a balun amplifier and a high-frequency amplifier [28–30], and the low frequency energy signals were derived from the anodes (Fig. 5b). The energy signal was split into two branches: one fed into the energy channel (ChxE), and the other directed to the timing channel (ChxT) of the BASP-10011 board. This configuration allows for the simultaneous acquisition of energy information through ADC and dual-threshold TOT. The energy value obtained by ADC is used as a reference only to evaluate the performance of dual-threshold TOT. The timing signals were directed into the other timing channels to capture the timestamp from the low threshold, while the high threshold functioned as a validation signal to minimize dead time.

Coincidence data were collected using a ^22^Na point source under controlled conditions, with the SiPM biased at 36 V and the experiment conducted at a stable temperature of 25 °C. For the dual-threshold TOT implementation (timing channel with energy signal), the low threshold was set at 50 mV, while the high threshold was set to 200 mV. Energy information from dual-threshold TOT was extracted using two methods. The first method involved direct estimation from pulse width, specifically the rise edge from the low threshold and the falling edge from the high threshold. The second method utilized curve fitting the scintillation pulse through bi-exponential modeling of four data points obtained through dual-threshold TOT. Subsequently, the energy information obtained through dual-threshold TOT was compared with the precisely measured energy values obtained together using an ADC. To evaluate linearity of dual-threshold TOT method, the estimated energy from TOT was compared with the energy obtained using an ADC. As an indicator of the linearity, INL was calculated as follows [22].

3 $$INL_{{100 - 600{\text{keV}}}} (E) = \frac{h(E) - f(E)}{{h(600{\text{keV}}) - h(100{\text{keV}})}}$$

4 $$INL_{\max } = \max [INL_{{100 - 600{\text{keV}}}} (E)]$$

5 $$INL_{\min } = \min [INL_{{100 - 600{\text{keV}}}} (E)]$$

6 $$INL_{mean} = \frac{{\left| {INL_{\max } } \right| + \left| {INL_{\min } } \right|}}{2}$$

where energy transfer curve *h(E)* is the energy acquired by using the dual-threshold TOT as a function of energy acquired with ADC, *f(E)* is the best-fitted straight line to *h(E)*. From the acquired data, *h(E)* was calculated at a 20 keV interval.

For timestamp pick-off (timing channel with timing signal), the high threshold remained constant at 200 mV, while the low threshold varied from 10 to 200 mV in increments of 10 mV to investigate the impact of varying threshold settings on time resolution performance.

## Results

### Threshold voltage of standard I/O

**Fig. 6:**
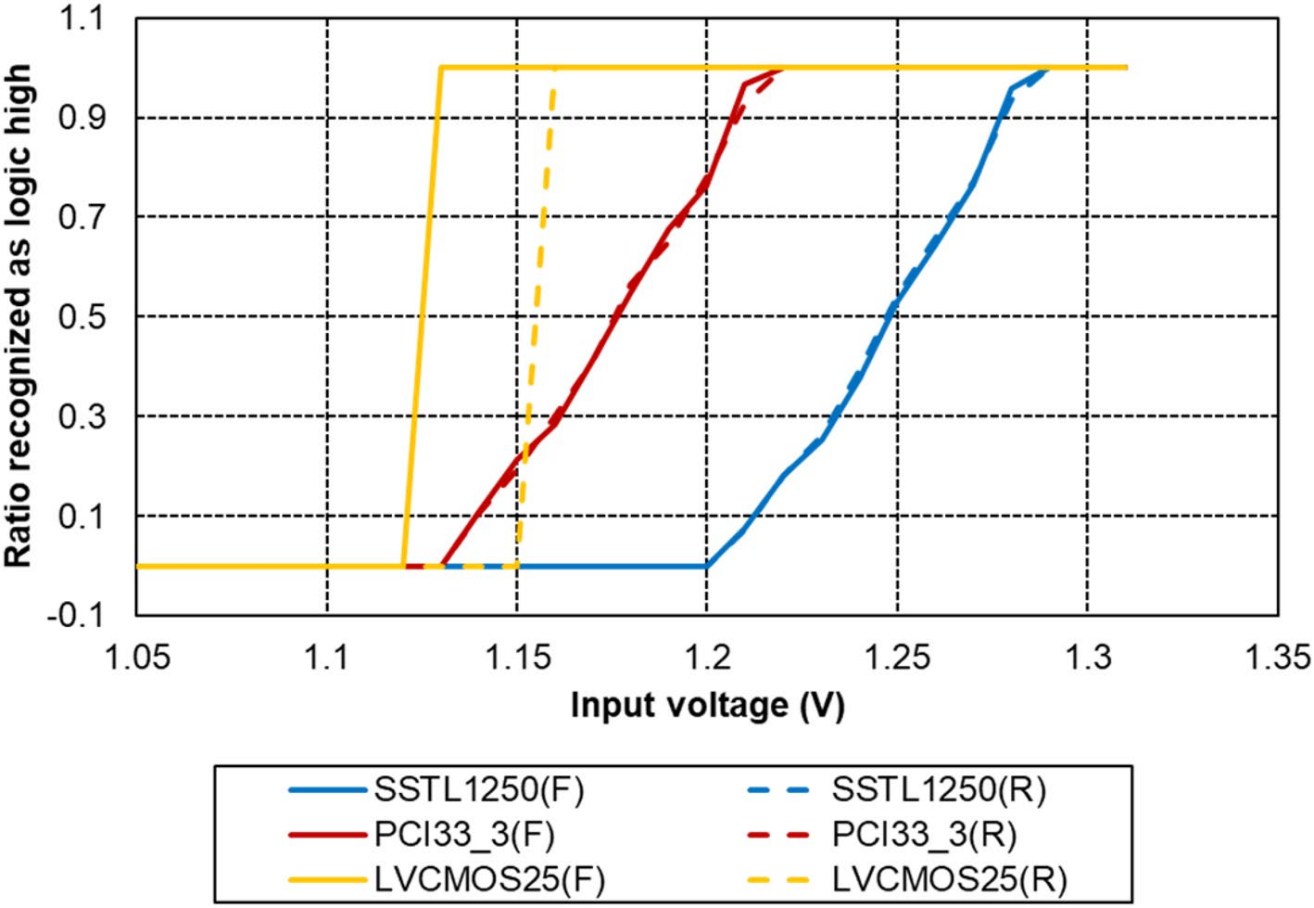
Experiment results to find the reference voltage of each IO standard

Figure 6 shows the reference voltage measurement results for three IO standards: SSTL with 1.25 V external reference voltage, PCI33_3, and LVCMOS25 with 2.5 V VCCO. In the cases of SSTL and PCI33_3, the ratio recognized as logic high reached 0.5 at the same threshold for both low-to-high and high-to-low transitions. However, for LVCMOS25, there was a disparity in the voltage levels at which the ratio recognized as logic high reached 0.5 during these transitions, resulting in a hysteresis of 30 mV. For the Artix-7 FPGA chip with 2.5 V VCCO, the reference voltage for PCI33 (*V*_*REF,PCI33*_) was 1.18 V, and the reference voltage for LVCMOS25 (*V*_*REF,LVCMOS25*_) was 1.125 V for high-to-low and 1.155 V for low-to-high transition.

As a consequence of this observation, in our study, we chose to utilize PCI33_3 in conjunction with SSTL for dual threshold implementation. This combination allowed us to implement the system effectively without the need for the correction to address the hysteresis issue in LVCMOS25.

### TDC performance

**Fig. 7:**
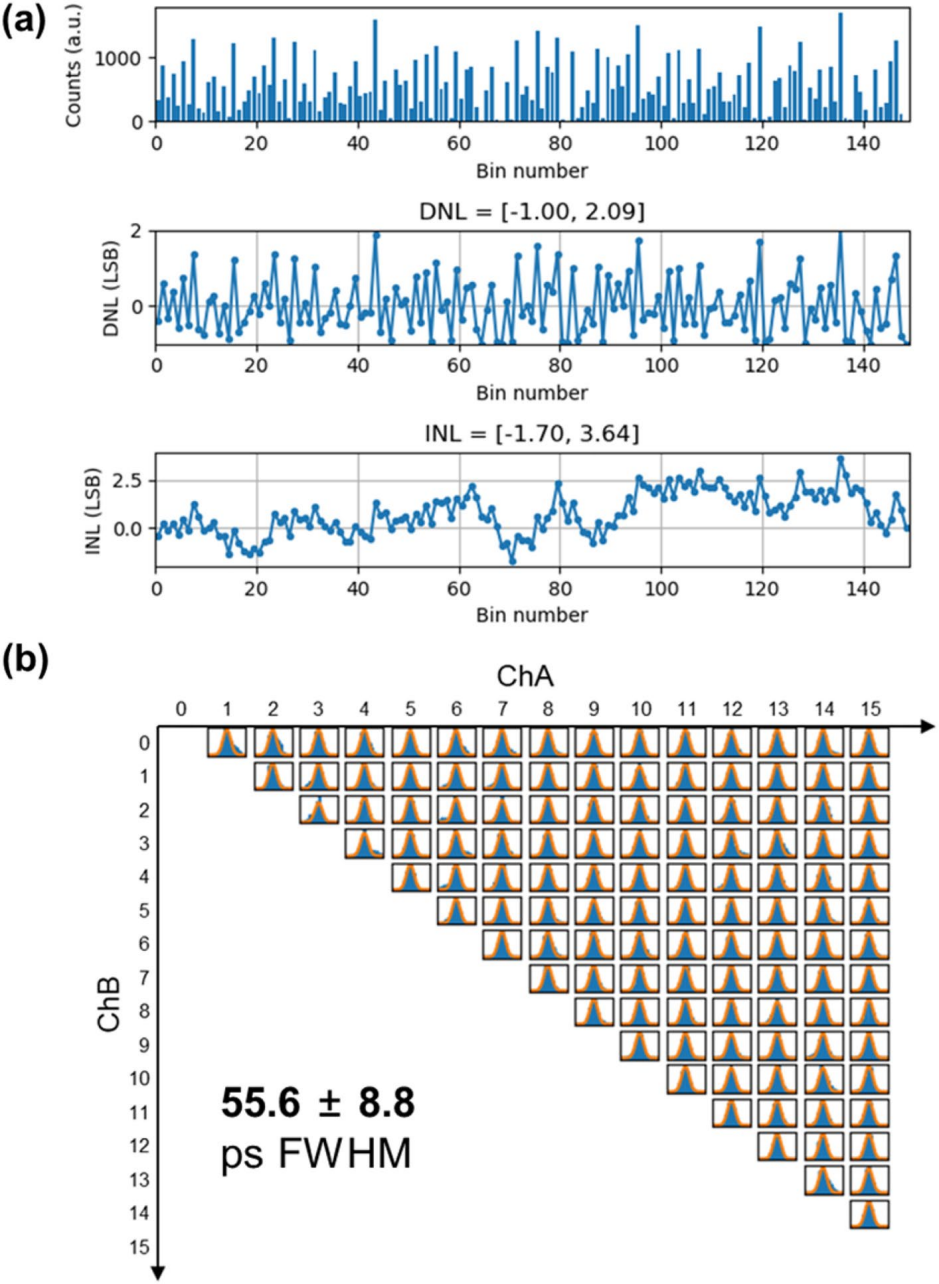
**a** Code density test, DNL and INL evaluation for representative TDC. **b** Time spectra for 120 TDC pair with identical pulse

Figure 7a shows the measurement results for the code density test, DNL, and INL of the representative TDC channel. The code density test revealed a maximum code of 151, corresponding to a resolution of 16.6 ps per least significant bit (LSB). Consistent with findings reported in various FPGA TDC studies [16, 17], the existence of a zero bin was observed, which impairs linearity due to the carry logic and clock network limitations inherent to the FPGA. Resulting DNL values were within [− 1.00, 2.09] LSB, and INL values were within [− 1.70, 3.64] LSB.

In Fig. 7b, the results of time resolution measurements for a combination of 120 different TDC pairs consisting of 16 TDCs are displayed, where identical square pulses were simultaneously applied to 16 channels. The average resolution across the 120 TDC pairs was determined to be 55.6 ± 8.8 ps full-width at half-maximum (FWHM), or equivalently, 23.6 ± 3.73 ps root mean square. The results demonstrate the intrinsic coincidence time resolution of the TDC pairs. Although the observed variances in the characteristics of the 16 TDC channels which is attributable to the dissimilarities in both internal and external routing pathways and the clock network depending on the TDC’s placement, an acceptable level of time resolution performance was realized across all channels. The intrinsic coincidence time resolution of 60 ps or less is suitable for modern TOF PET systems, achieving a time resolution of less than 200 picoseconds.

### Detector performance measurement

**Fig. 8:**
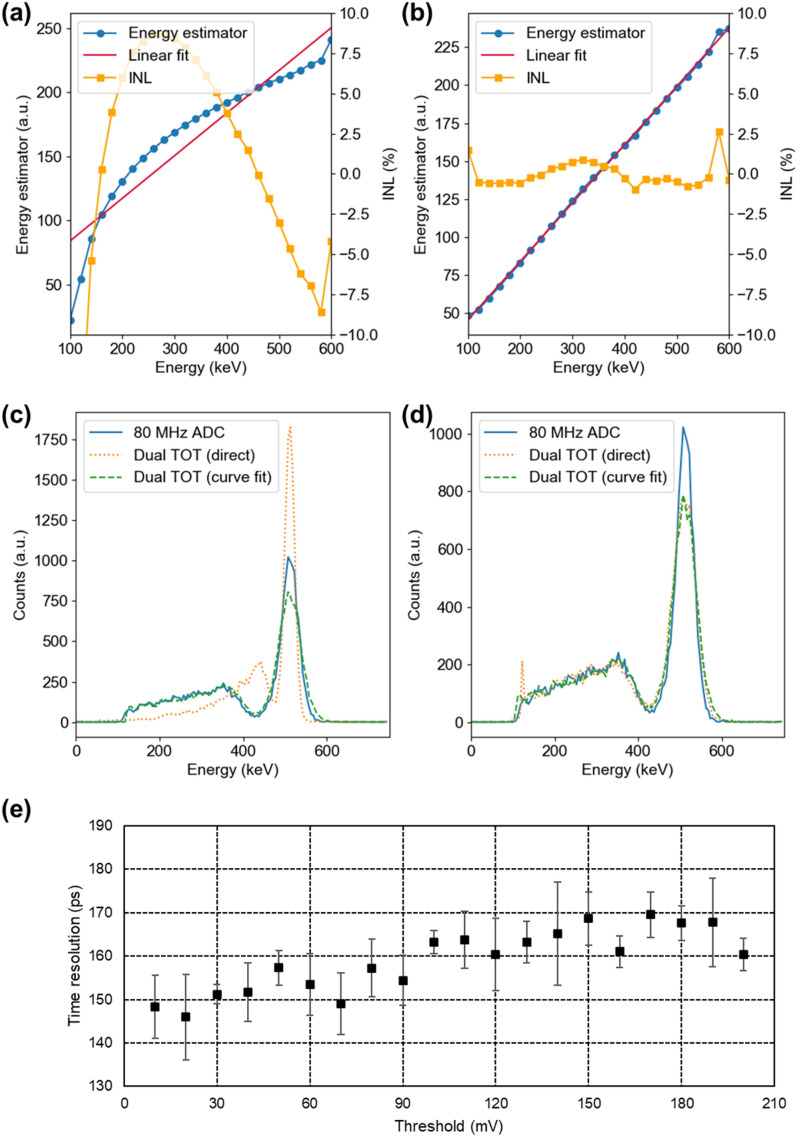
Energy linearity and INL plot for dual-threshold TOT **a** with direct estimation and **b** curve fitting method. Energy spectra for ADC and dual-threshold TOT **c** without linearity correction and **d** with linearity correction. **e** Time resolution measurement results according to the threshold voltage

To demonstrate the usefulness of the developed technology for TOF PET application, the performance of one-to-one coupled TOF PET detector were measured. Figure 8a and b shows the energy dependent INL for dual-ended TOT with direct estimation and curve fitting, respectively. The results demonstrate favorable linearity within the energy range of 100 keV to 600 keV, with *INL*_min_ = − 1.74%, *INL*_max_ = 1.68%, and *INL*_*mean*_ = 1.71% for curve fitting method. Direct estimation showed lower linearity with with *INL*_min_ = − 13.7%, *INL*_max_ = 6.06%, and *INL*_*mean*_ = 9.88%.

Figure 8c and d illustrates the energy spectra acquired from a single scintillation crystal detector before and after energy linearity correction, respectively. Thanks to its good linearity, the dual-threshold TOT with curve fitting method exhibit strong agreement with the ADC results, even without the linearity correction. Conversely, the dual-ended TOT with direct estimation initially produced a skewed energy spectrum. However, following linearity correction, it achieved satisfactory energy resolution. The energy resolution was measured at 10.8% when employing ADC and slightly higher, at 12.5% and 12.8%, when using the dual-threshold TOT method with curve fitting and direct estimation.

In Fig. 8e, we explore the effects of changing the low threshold while keeping the high threshold of the timing channel fixed at 200 mV. As the low threshold increased from 10 to 200 mV in 10 mV increments, the time resolution deteriorated. The optimal time resolution, recorded at 146 ± 9 ps FWHM, was achieved under conditions with a low threshold of 20 mV. Even at a low threshold of 10 mV, a time resolution of under 150 ps was maintained, without a steep increase in timing resolution. This can be attributed to the strategy of utilizing the high threshold as a validation signal, effectively minimizing the impact of false triggers.

When data were collected using an oscilloscope (MSOS404A, Keysight, USA) under the same detector and threshold conditions, a result of 124 ± 4 ps was achieved. It's worth noting that the results obtained with the FPGA-only digitizer showed a slight degradation compared to this, which can be anticipated, given the TDC’s intrinsic resolution of the developed digitizer, which stands at 55.6 ps.

## Discussion

As SiPM replaced the conventional photomultiplier tube in the PET system, the number of photosensors compared to scintillation crystals significantly increased. The increase in the axial lenght of the PET system and the advanced SiPM signal readout technologies to achieve better timing resolution are other causes that increase the number of photosensors and readout complexity. To address this issues, in this study, we introduced an efficient methodology for implementing a dual-threshold input receiver utilizing commercially available FPGAs. Our approach minimizes the number of FPGA IO pins required and reduces routing complexity, thereby simplifying PCB design. This method is particulary beneficical for the development of sophisticated multi-channel DAQ systems with minimal circuit complexity and resource usage. To demonstrate the usefulness of the proposed technology in PET digitizer application, a dual-threshold input receiver was used for implementing TOT to obtain energy information and validation signals to reduce deadtime. With this configuration, we achieved an energy resolution of 12.5% and a time resolution of 146 ps in a 20 mm long scintillation crystal using only the FPGA. The results demonstrate that the proposed FPGA-only digitizer concept has good potential to simplify the complex signal processing of modern TOF PET.

For proof-of-concept, we used the cost-effective Artix-7 FPGA. Although time resolution can be improved by upgrading to newer advanced FPGAs such as the Ultrascale or Ultrascale + series, this study has shown that Artix-7 FPGAs can adequately support TOF PET systems with a time resolution of 150 ps. Considering the high cost per channel of these advanced FPGAs for one-to-one readout applicaions, the fact that sufficient performance can be achieved using the Artix-7 FPGA is encouraging. Moreover, should further cost reductions be necessary, Spartan series FPGAs are a viable alternative. Although not demonstrated in this study, it’s anticipated that the suggested method could be applicable to FPGAs from Altera or Lattice, which offer HSTL, SSTL, and other digital interfaces.

Contrary to the charge-to-digital converter (QTC) approach [23, 26, 31], widely used for enhancing energy linearity in time-based energy readout, the dual threshold TOT used in this paper has the advantage of minimizing dead time and circuit complexity. The QTC necessitate supplementary components like charge amplifiers for the capacitor’s charge and discharge cycle. This not only extends dead time but also requires extra control circuits to control these processes, complicating the overall circuit design.

The curve fitting method using the bi-exponential function has the advantage of being able to estimate energy information accurately with high linearity, but has the disadvantage of complicated calculation. One alternative to reduce this complexity is curve fitting using the linear least square method. As the method proposed in STS research [22], the non-linear bi-exponential function used in curve fitting can be converted to a linear function by using the fact that the rise and decay time of the scintillation signal are constant. This linear fitting can be easily implemented with the FPGA. In addition to the concept of FPGA-only digitizer shown in this paper, there is a way to further improve the performance of PET detectors using the proposed dual threshold implementation method. For example, two timestamps can be utilized to more accurately estimate the photon arrival time. In particular, a dual threshold can be used to measure the rise time of the input signal to improve the time resolution of the bismuth germanate (BGO) Cherenkov detector. In the BGO detector, time resolution can be improved by classifying timestamps based on the rise time of the input signal [30, 32]. In addition, machine learning or deep learning techniques that use the multiple timestamps as input may also contribute to improving the time resolution of the BGO detector.

In our demonstration, we implemented a dual threshold using SSTL and PCI33 IO standards. However, given the distinct reference voltages of PCI33 and LVCMOS, it is feasible to incorporate more than three threshold voltages by integrating additional IO standards. Furthermore, other IO standards not evaluated in this study could also be considered. These method to configure three or more thresholds is particulary advantageous for implementing the MVT method [20, 21]. The MVT approach requires the use of additional IO resources but allows for more accurate estimation of energy information.

Although the reference voltage measurements of the IO standard revealed hysteresis issues associated with the LVCMOS IO standard, it is noteworthy that this standard exhibited a more rapid response during transitions. In this study, we chose not to employ the LVCMOS IO standard to avoid the complexities associated with hysteresis. Nonetheless, future investigations are needed to evaluate potential differences in time resolution between the LVCMOS and SSTL/PCI33 interfaces, providing a comprehensive understanding of their comparative performance.

## Conclusion

The proposed method enables the configuration of an FPGA-only digitizer with increased efficiency. This advancement holds the potential to greatly assist in the miniaturization of TOF PET electronics.
